# Supplementary material for: Single-Cell RNA-Seq Reveals Transcriptomic Heterogeneity and Post-Traumatic Osteoarthritis-Associated Early Molecular Changes in Mouse Articular Chondrocytes
Source: Cells. 2021 Jun 10;10(6):1462. doi: 10.3390/cells10061462 (PMC8230441; doi:10.3390/cells10061462)
Supplement: Supplementary file 1 [file cells-10-01462-s001.zip › cells-1229572-supplementary.pdf]

Single-Cell RNA-Seq Reveals Transcriptomic Heterogeneity and Post-Traumatic Osteoarthritis-Associated Early Molecular Changes in Mouse Articular Chondrocytes

Aimy Sebastian, Jillian L. McCool, Nicholas R. Hum, Deepa K. Murugesh , Stephen P. Wilson ,
Blaine A. Christiansen and Gabriela G. Loots

Supplementary Figures

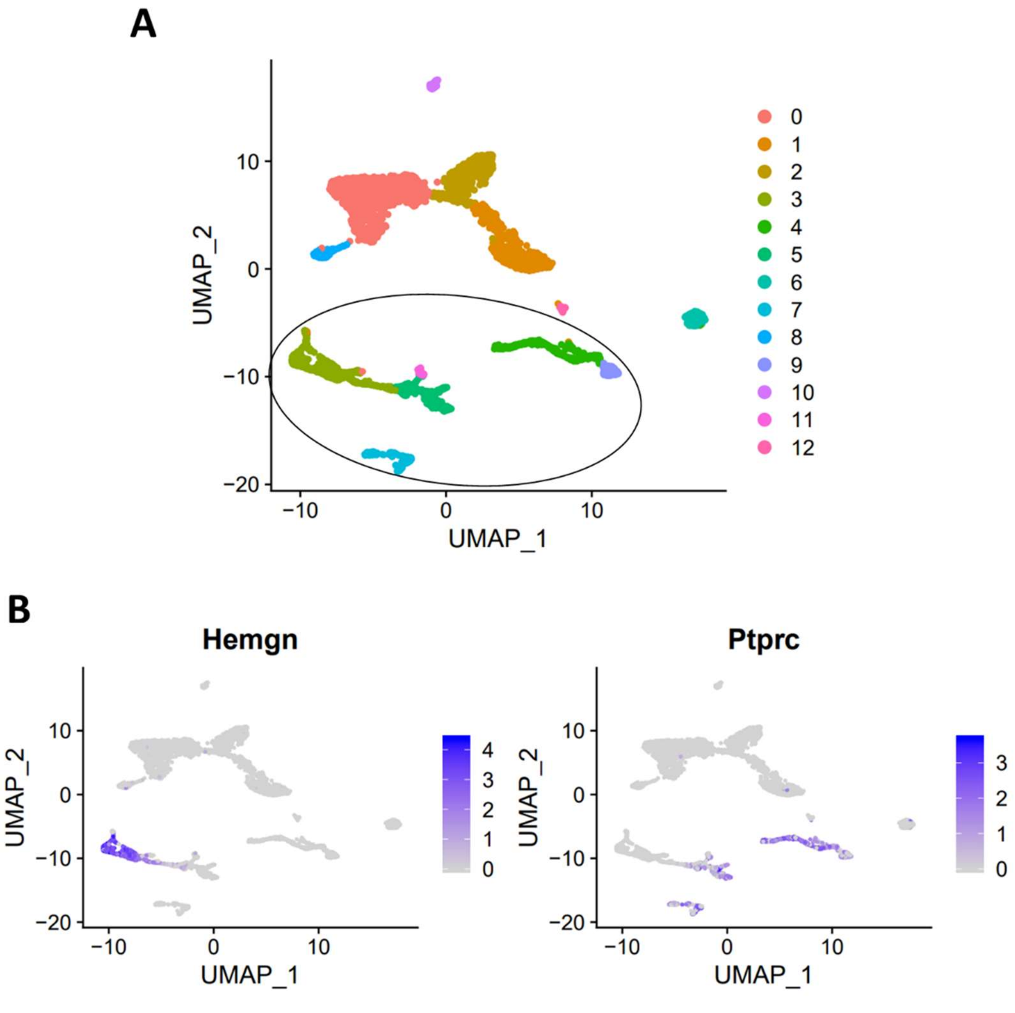

***Figure S1**: **Single-cell analysis of 10-week-old BL6 mouse knee joints.** A) Cell clusters from scRNA-seq analysis visualized by Uniform Manifold Approximation and Projection (UMAP). Colors indicate clusters of various cell types. Immune and blood cell clusters are in black oval. B) Feature plot showing the expression of erythroid cell marker Hemgn and immune marker Ptprc (CD45).*

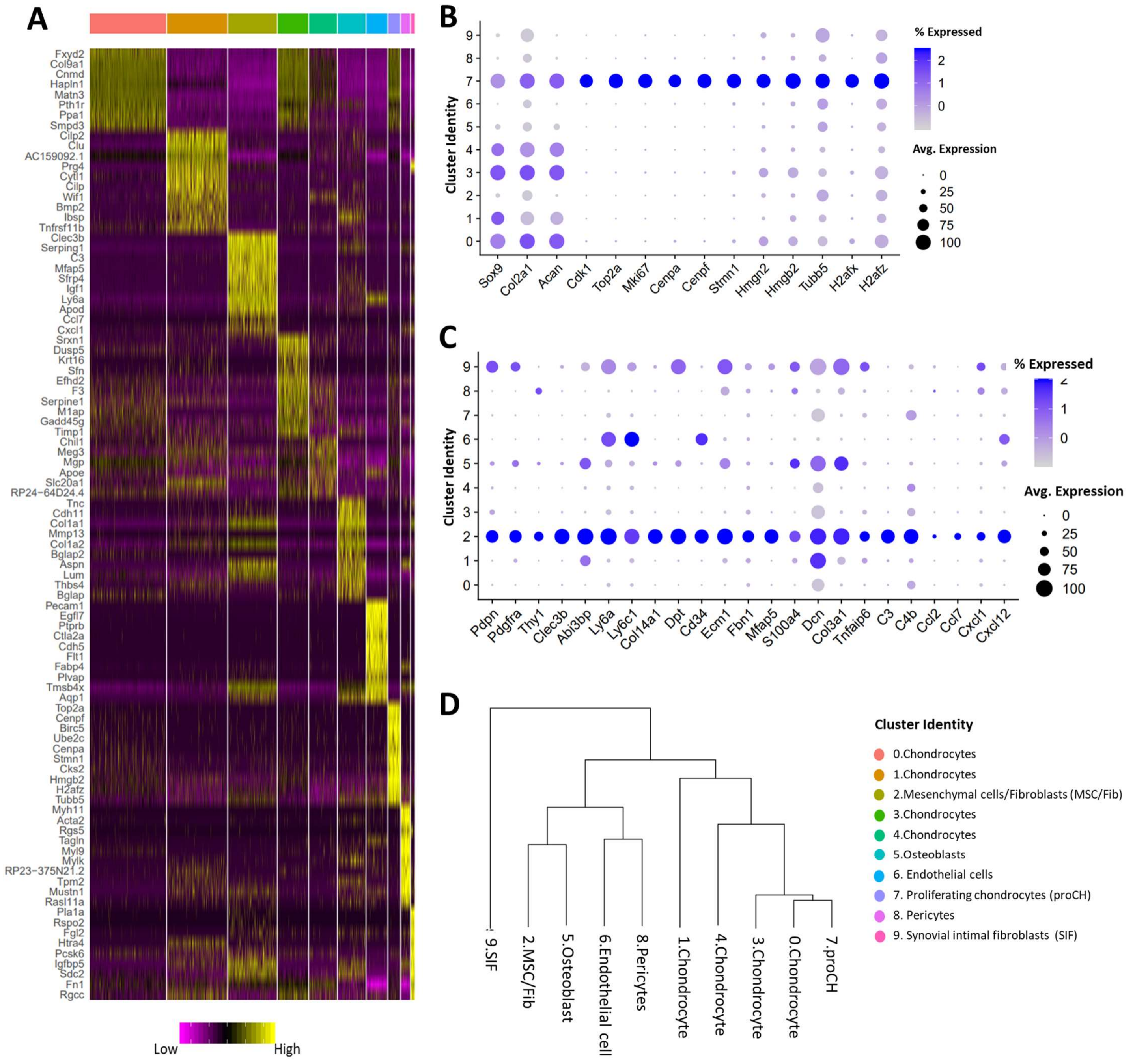

***Figure S2**: **Characterization of connective tissue-forming cells in the joint**. A) Heatmap showing top markers of various connective-tissue forming cell clusters. B) Dot plot showing the expression of selected cell cycle genes. Dot size represents the fraction of cells expressing a specific marker in a particular cluster and intensity of color indicates the average expression level in that cluster. C) Dot plot showing the expression of selected fibroblast/mesenchymal cell markers. D) A cluster tree showing the relationship between various clusters.*

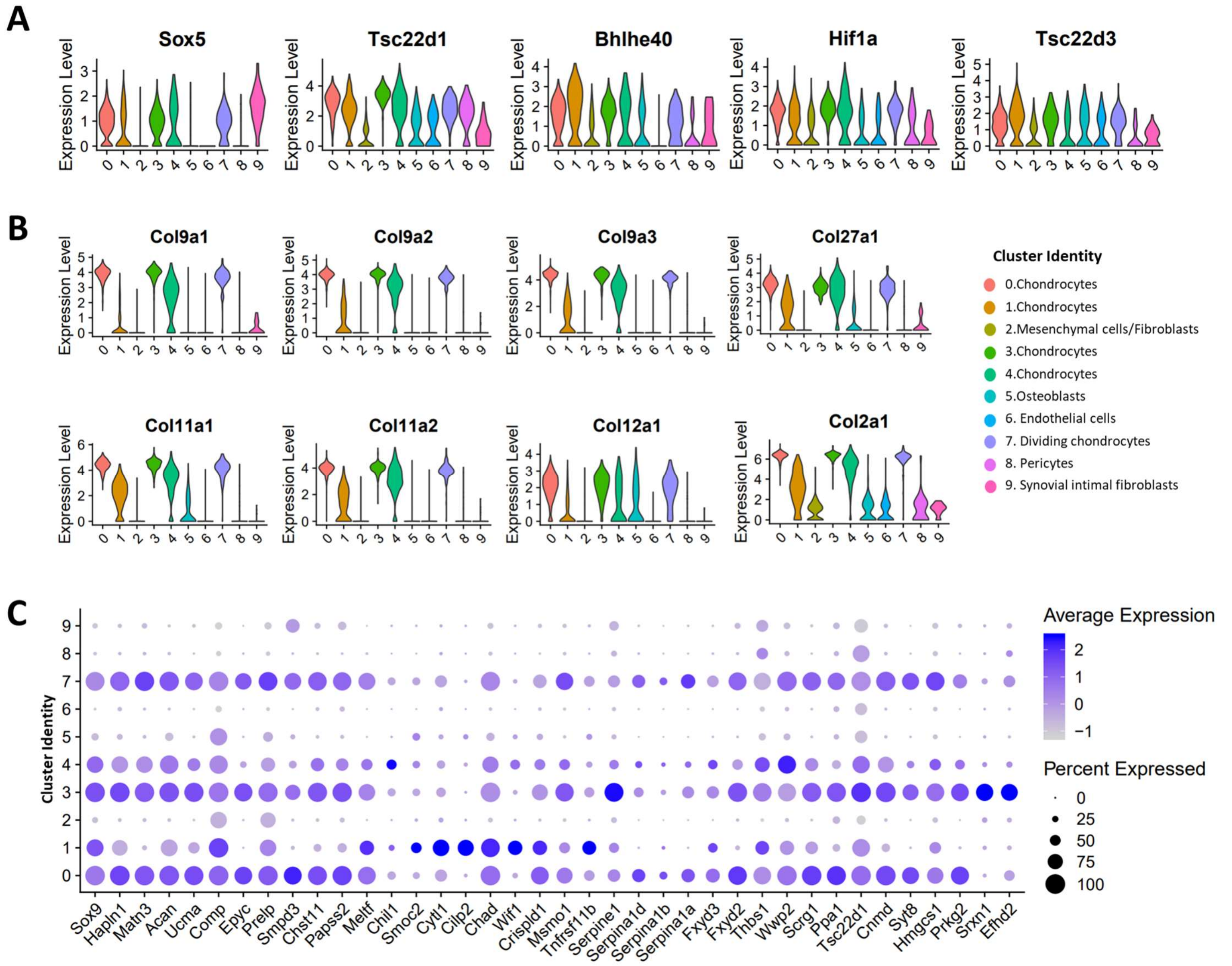

Figure S3*: *Comparison of chondrocytes to other connective tissue-forming cells. *A) Violin plot showing the expression of selected transcription factors enriched in chondrocyte clusters. B) Violin plot showing the expression of collagens enriched in chondrocyte clusters. C) Dot plot showing the expression of selected genes enriched in chondrocyte clusters compared to other connective tissue-forming cells. Dot size represents the fraction of cells expressing a specific marker in a particular cluster and intensity of color indicates the average expression level in that cluster.*

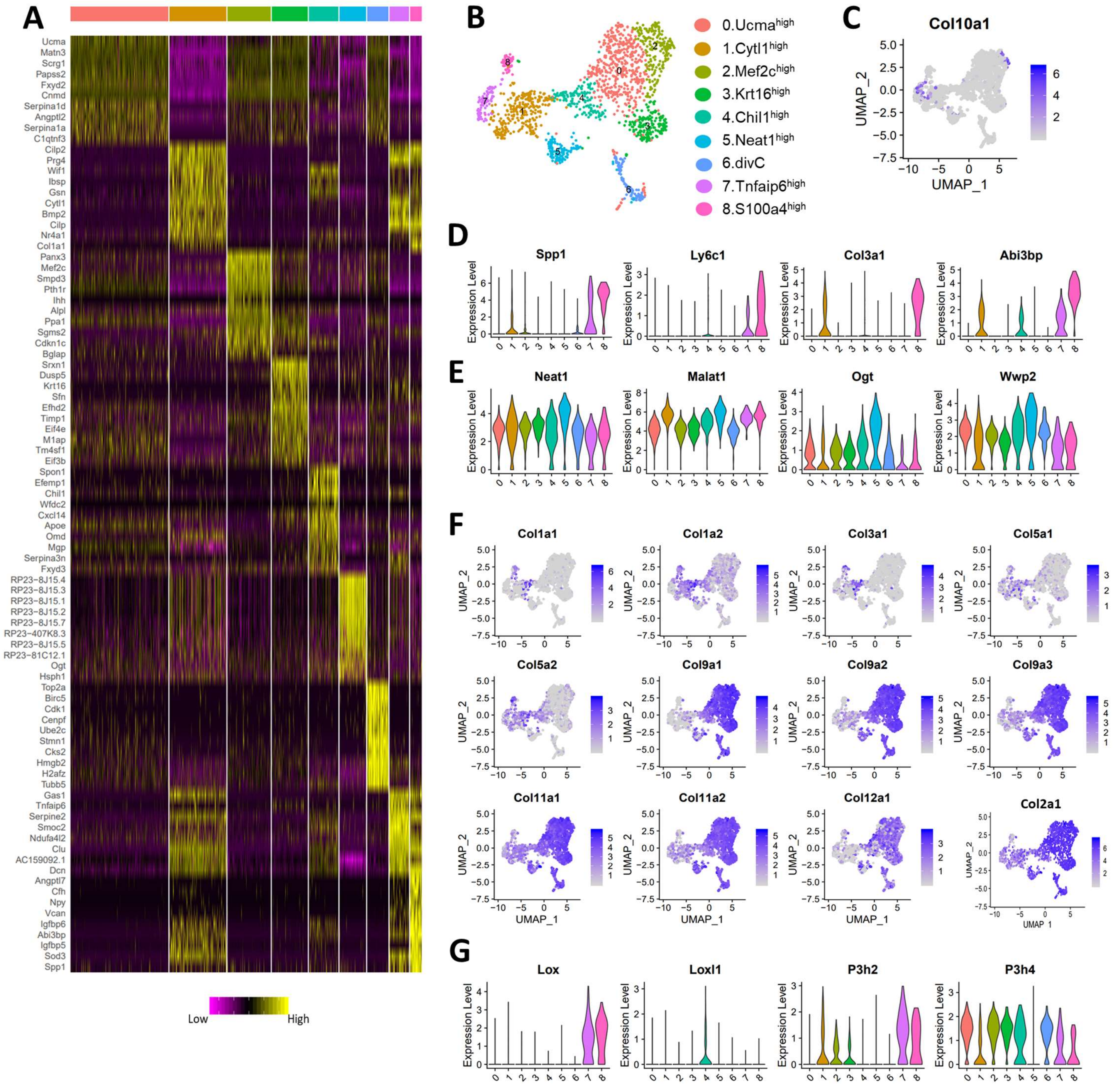

***Figure S4*: *Characterization of chondrocyte subpopulations*.** *A) Heatmap showing top markers of various chondrocyte subclusters. B) UMAP plots of various chondrocyte subclusters. Colors indicate clusters of various cell types with distinct gene expression profiles. C) Feature plot showing the expression of Col10a1. D) Violin plot showing the expression of selected fibroblast/fibrosis markers enriched in cluster 8. E) Violin plot showing the expression of a subset of genes enriched in cluster 5. F) Feature plot showing the expression of various collagens in chondrocyte subclusters. D) Violin plot showing the expression collagen processing enzymes in chondrocyte subclusters.*

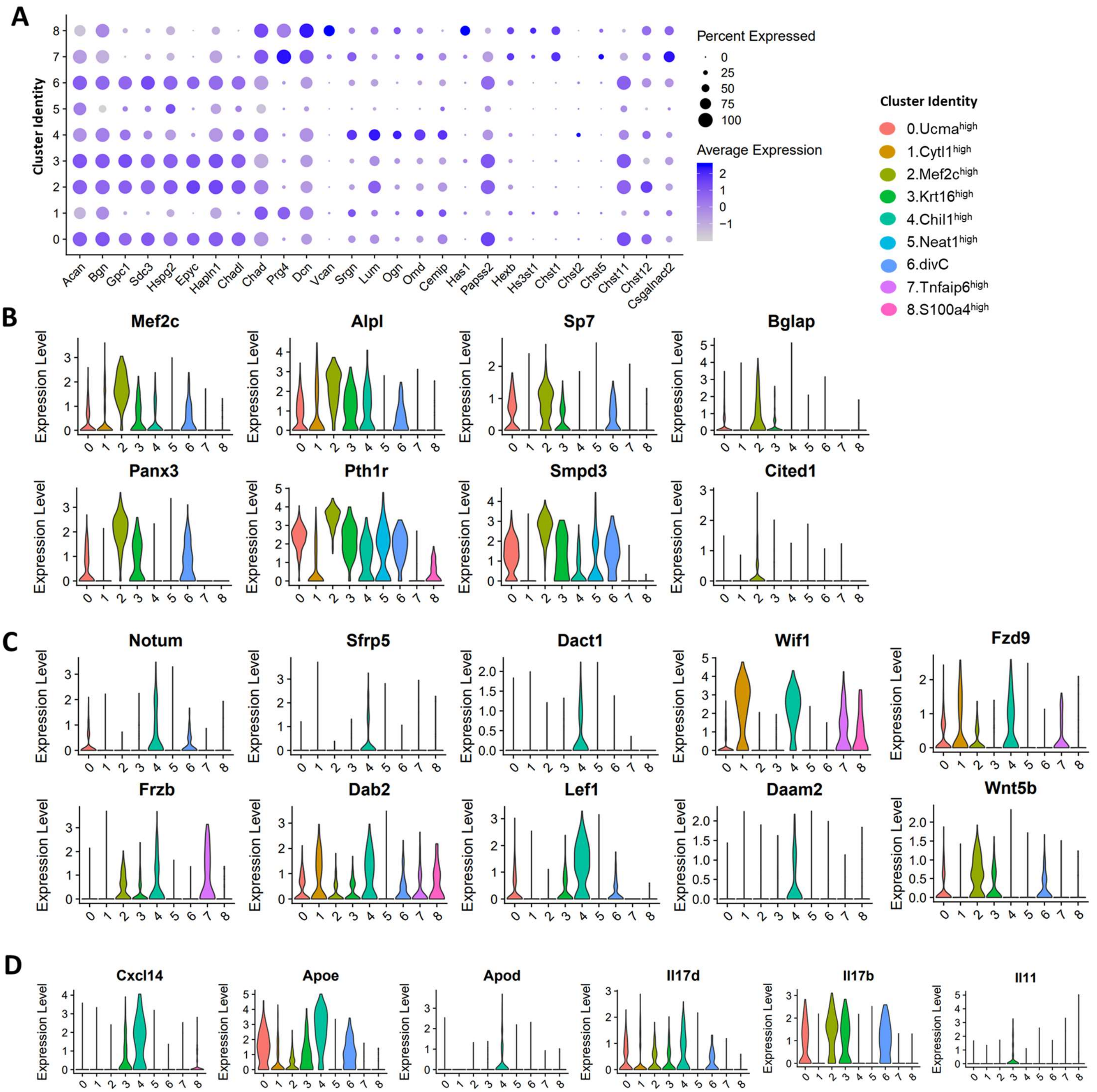

Figure S5**: **Proteoglycans and Wnt pathway genes are differentially expressed between chondrocyte subpopulations. *A) Dot plot showing the expression of selected proteoglycans and proteoglycan processing enzymes. Dot size represents the fraction of cells expressing a specific marker in a particular cluster and intensity of color indicates the average expression level in that cluster. B) Violin plot showing the expression of biomineralization-related genes enriched in Mef2c^{high} cluster. C) Violin plot showing the expression of Wnt signaling pathway genes. D) Violin plot showing the expression of selected secreted signaling proteins.*

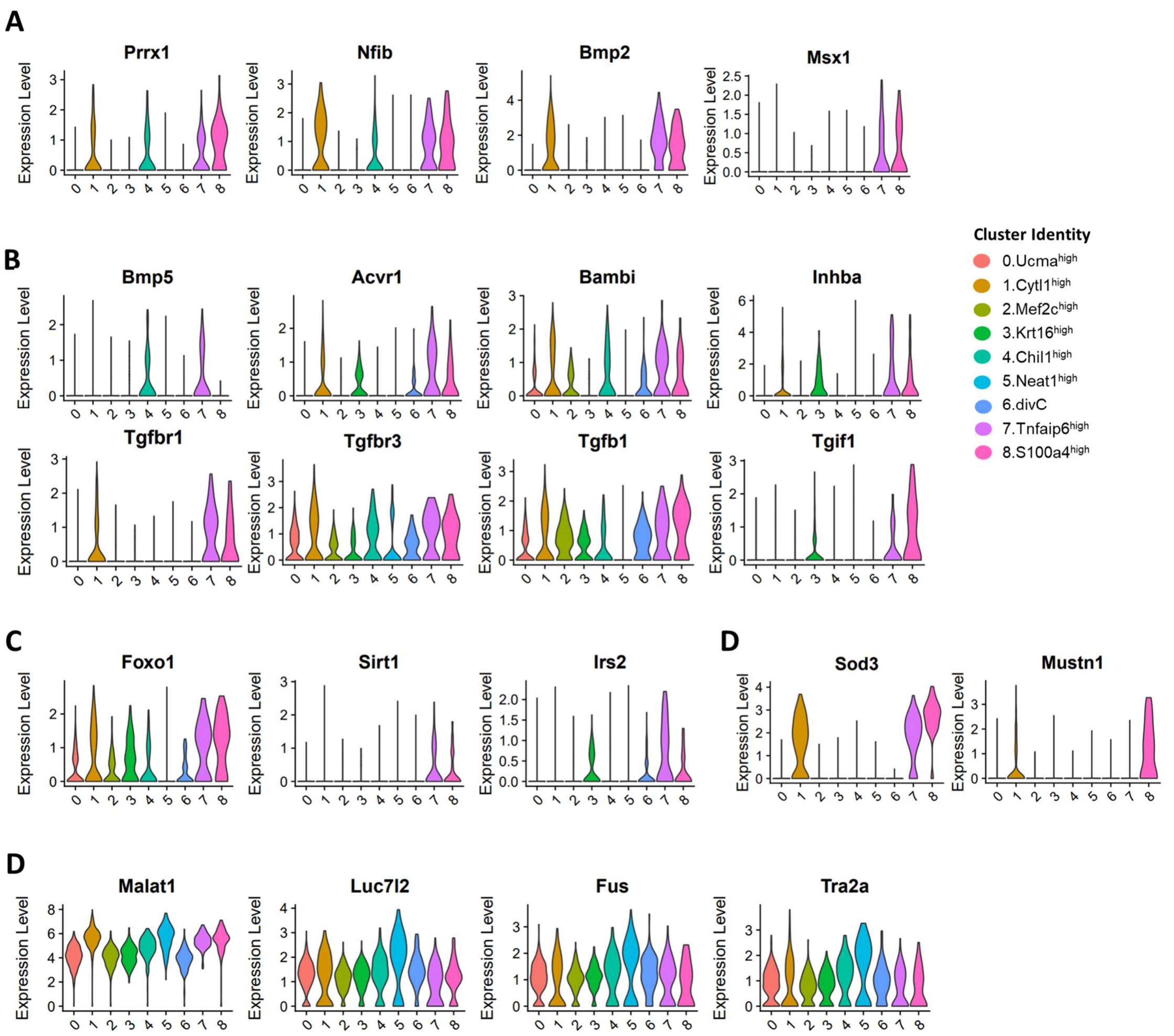

***Figure S6**: **TGFβ/BMP pathway genes and regulators of mesenchymal cell proliferation are differentially expressed between chondrocyte subpopulations**. A) Violin plot showing the expression of mesenchymal cell proliferation-related genes. B) Violin plot showing the expression of TGFβ/BMP signaling pathway genes. C) Violin plot showing the expression of FOXO signaling-related genes. D) Violin plot showing the expression of selected genes enriched in S100a4^{high} cluster. E) Violin plot showing the expression of RNA processing and splicing genes.*

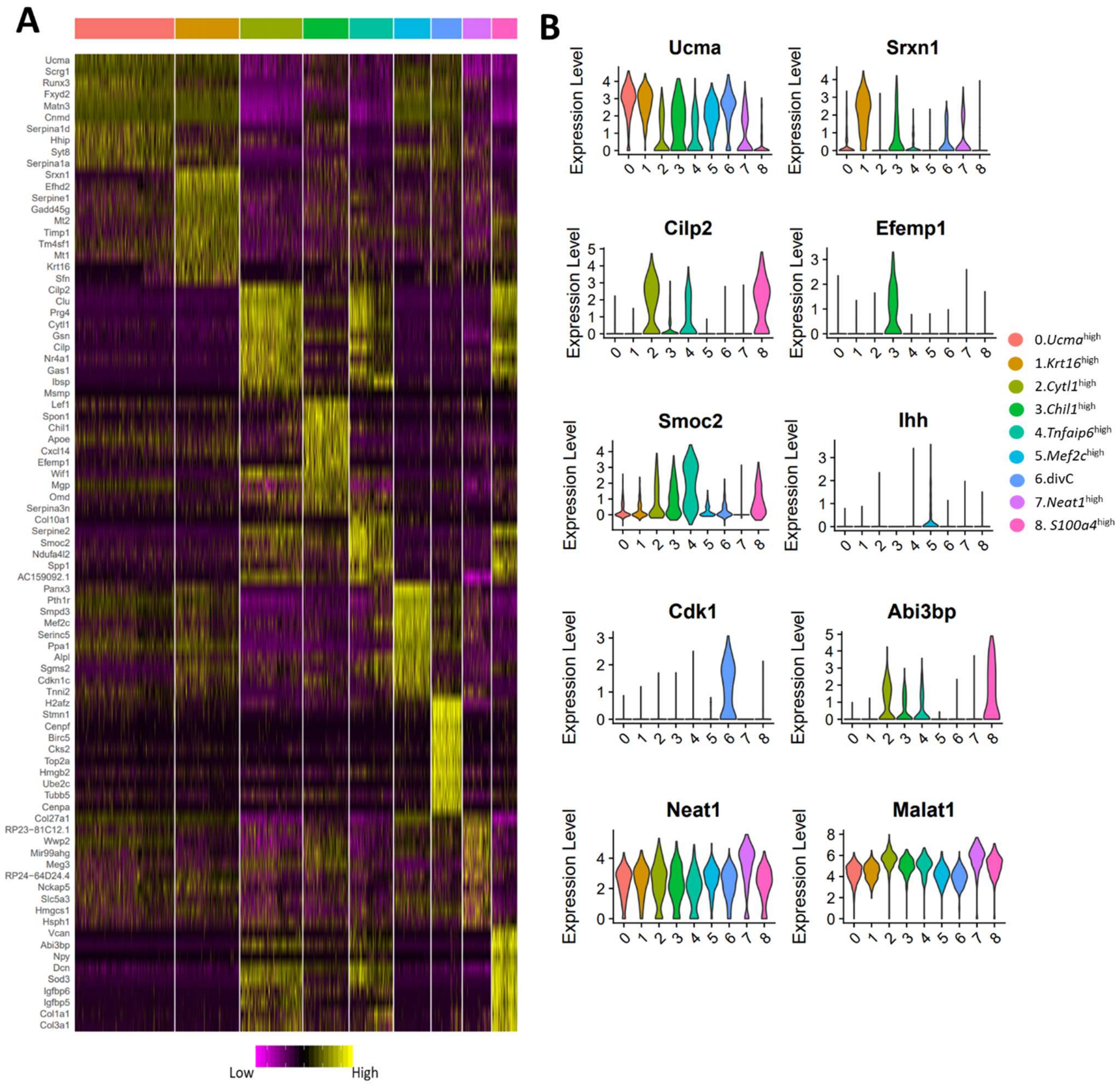

***Figure S7**: **Characterization of chondrocyte subpopulations from injured and uninjured knee joints**. A) Heatmap showing top markers of various chondrocyte subclusters. B) Violin plot showing the expression of selected chondrocyte subtype markers.*

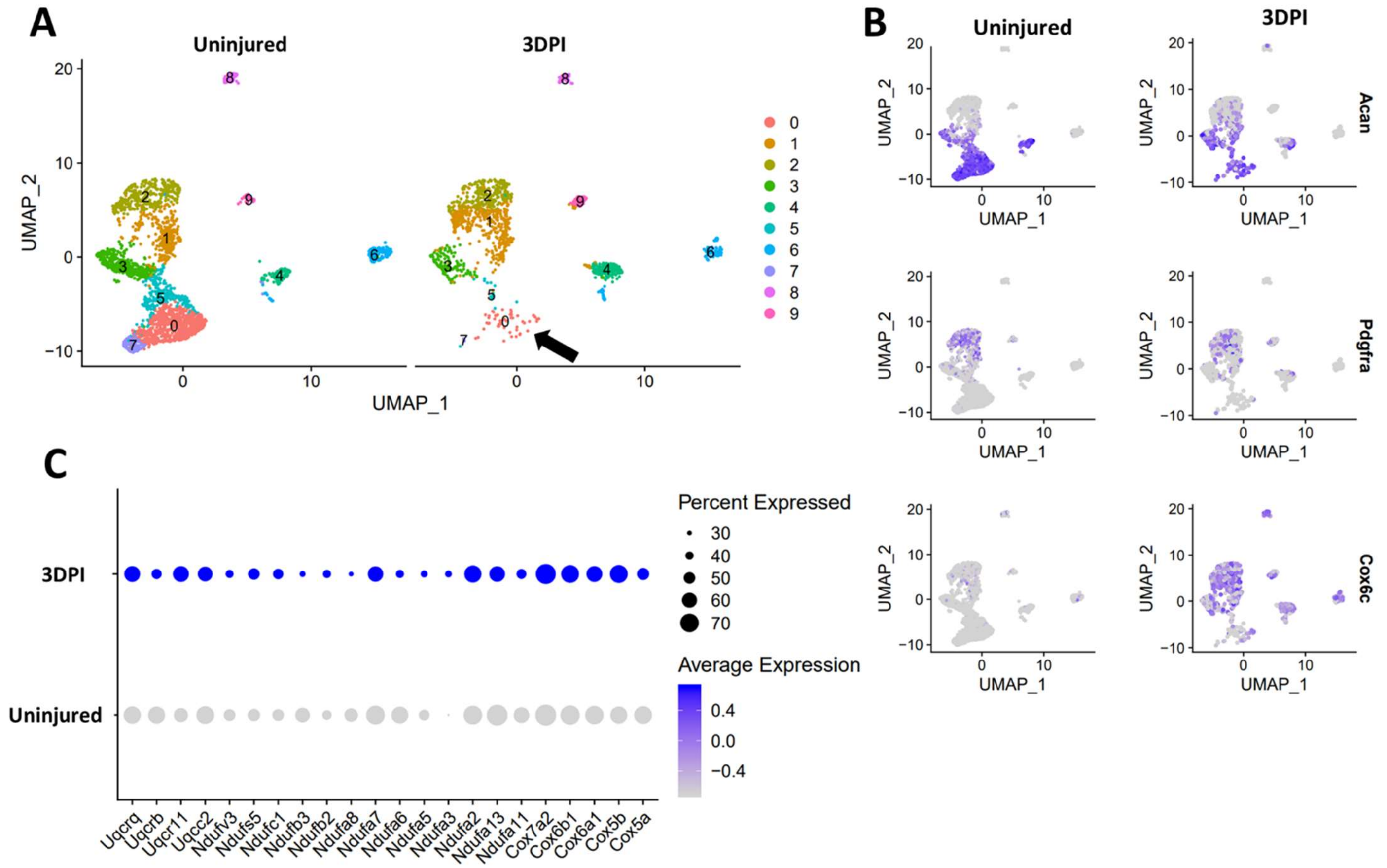

***Figure S8**: **Cellular and molecular changes at 3 days post-injury (3DPI)**. A) UMAP plot of different connective tissue forming cell types. Chondrocyte clusters showing significant reduction in cell number are highlighted with black arrow. B) Feature plot showing the expression of chondrocyte marker Acan, fibroblast marker Pdgfra and Cox6c. C) Dot plot showing the average expression of selected OXPHOS enzymes in all connective tissue forming cells from uninjured and 3DPI joints.*

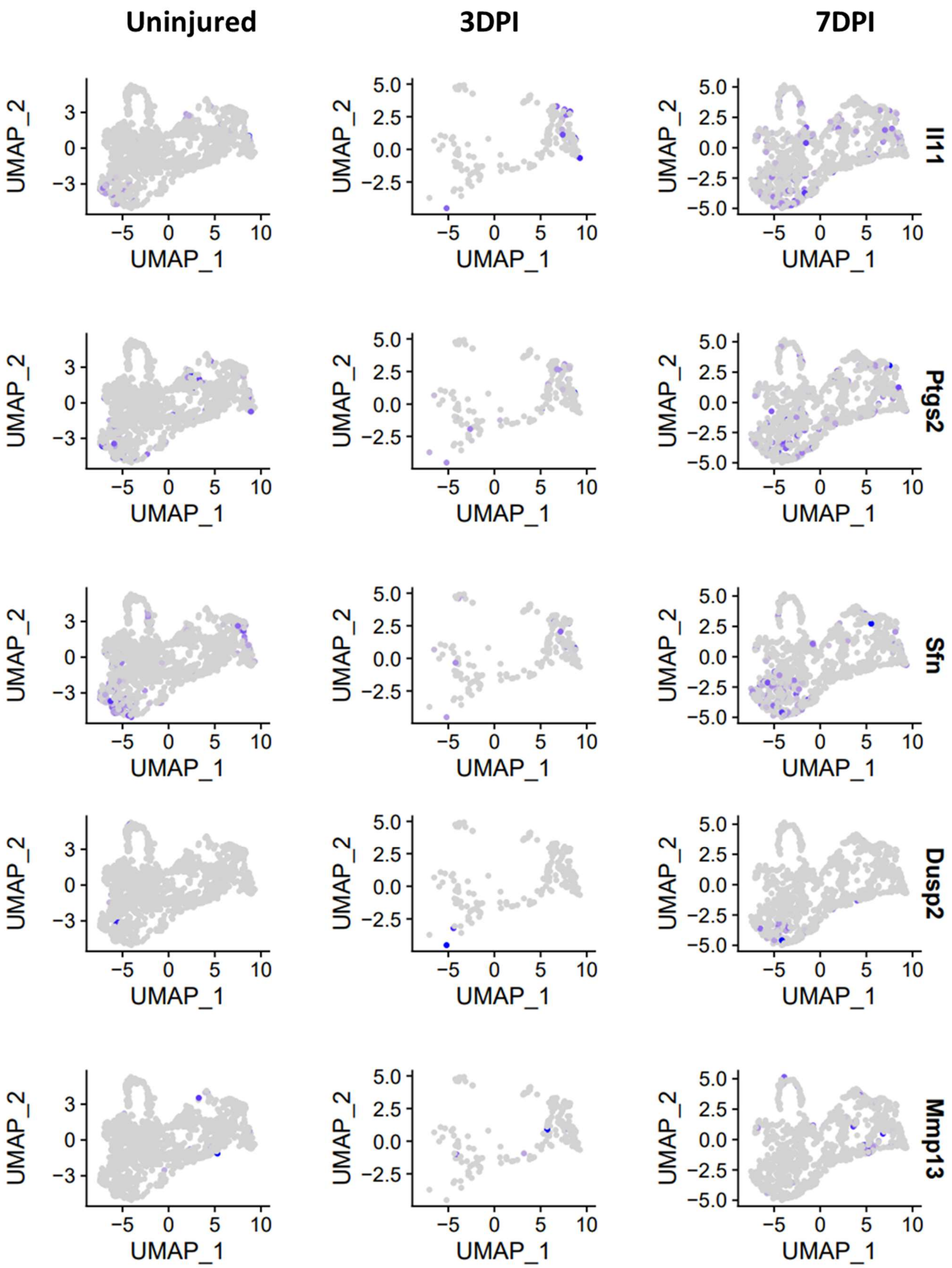

Figure S9: ***Genes upregulated after injury***. *Feature plots showing the expression of selected genes upregulated after injury.3DPI: 3 days post-injury. 7DPI: 7 days post-injury.*

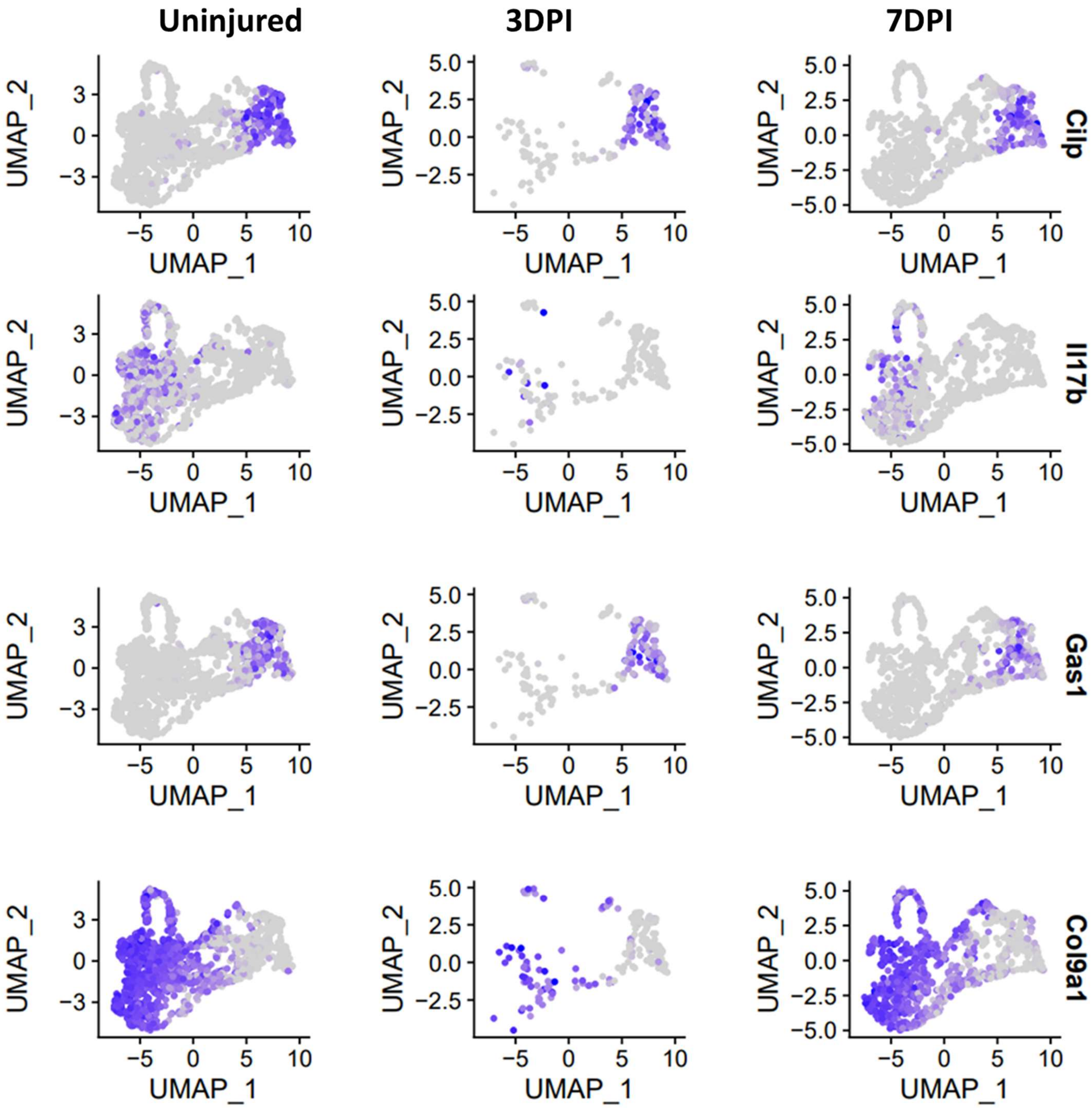

***Figure S10**: **Genes downregulated after injury**. Feature plots showing the expression of selected genes downregulated after injury.3DPI: 3 days post-injury. 7DPI: 7 days post-injury.*

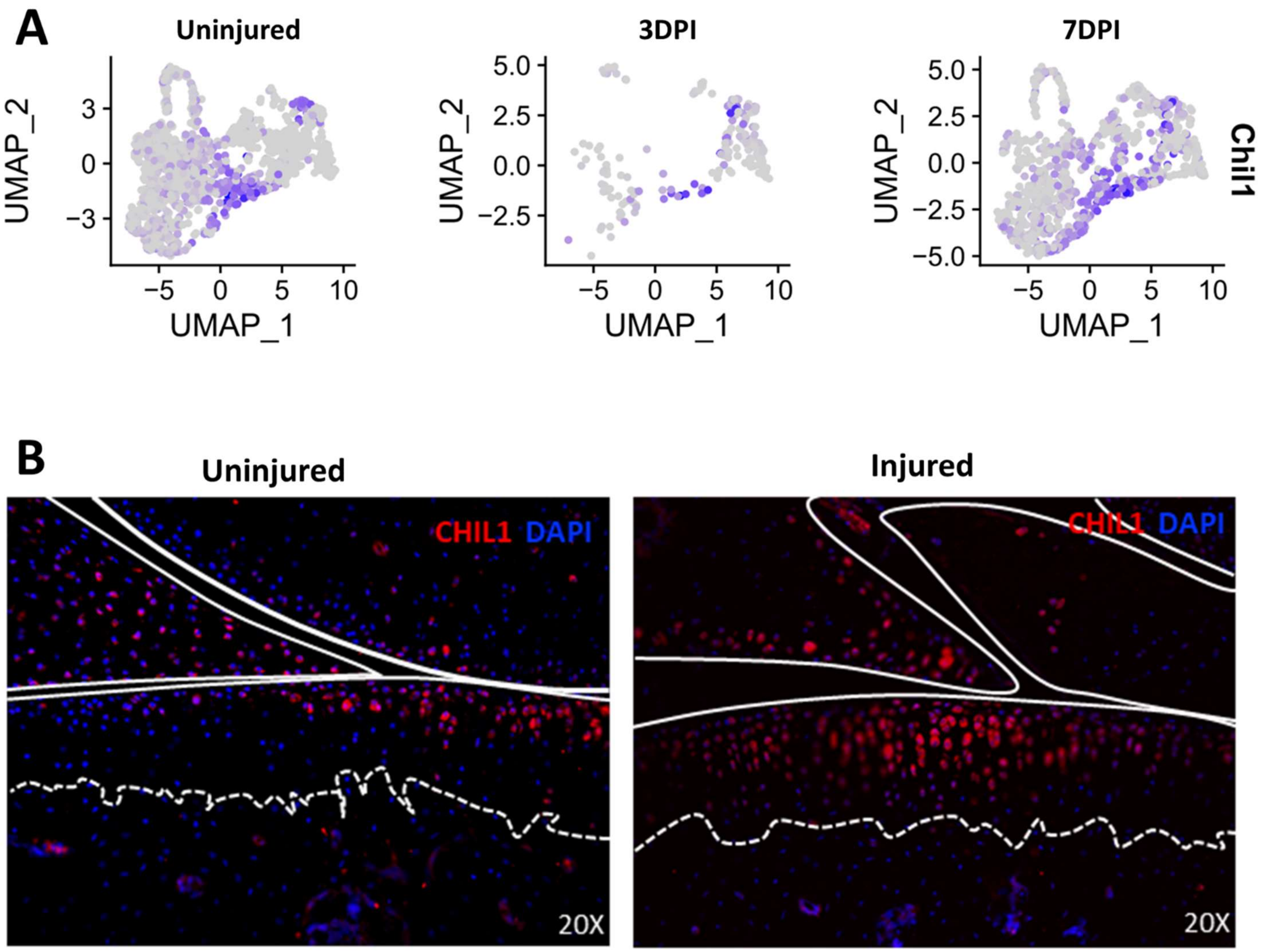

***Figure S11**: **Expansion of Chil1 expressing region after injury**. A) Feature plots showing the expression of Chil1 trancript.3DPI: 3 days post-injury. 7DPI: 7 days post-injury. B) Protein-level expression of Chil1.*
